# A prediction model for short-term neurodevelopmental impairment in preterm infants with gestational age less than 32 weeks

**DOI:** 10.3389/fnins.2023.1166800

**Published:** 2023-04-24

**Authors:** Yan Li, Zhihui Zhang, Yan Mo, Qiufen Wei, Lianfang Jing, Wei Li, Mengmeng Luo, Linxia Zou, Xin Liu, Danhua Meng, Yuan Shi

**Affiliations:** ^1^Department of Neonatology, Children’s Hospital of Chongqing Medical University, National Clinical Research Center for Child Health and Disorders, Ministry of Education Key Laboratory of Child Development and Disorders, Chongqing Key Laboratory of Pediatrics, Chongqing, China; ^2^Department of Applied Mathematics, The Hong Kong Polytechnic University, Kowloon, Hong Kong SAR, China; ^3^Neonatal Medical Centre, Maternal and Child Health Hospital of Guangxi Zhuang Autonomous Region, Nanning, China; ^4^Guangxi Clinical Research Center for Pediatric Diseases, Nanning, China; ^5^Department of Biological Sciences, University of Liverpool, Liverpool, United Kingdom

**Keywords:** gestational age less than 32 weeks, prematurity, neurodevelopmental impairment, support vector machine, principal component analysis

## Abstract

**Introduction:**

Early identification and intervention of neurodevelopmental impairment in preterm infants may significantly improve their outcomes. This study aimed to build a prediction model for short-term neurodevelopmental impairment in preterm infants using machine learning method.

**Methods:**

Preterm infants with gestational age  < 32 weeks who were hospitalized in The Maternal and Child Health Hospital of Guangxi Zhuang Autonomous Region, and were followed-up to 18 months corrected age were included to build the prediction model. The training set and test set are divided according to 8:2 randomly by Microsoft Excel. We firstly established a logistic regression model to screen out the indicators that have a significant effect on predicting neurodevelopmental impairment. The normalized weights of each indicator were obtained by building a Support Vector Machine, in order to measure the importance of each predictor, then the dimension of the indicators was further reduced by principal component analysis methods. Both discrimination and calibration were assessed with a bootstrap of 505 resamples.

**Results:**

In total, 387 eligible cases were collected, 78 were randomly selected for external validation. Multivariate logistic regression demonstrated that gestational age(*p* = 0.0004), extrauterine growth restriction (*p* = 0.0367), vaginal delivery (*p* = 0.0009), and hyperbilirubinemia (0.0015) were more important to predict the occurrence of neurodevelopmental impairment in preterm infants. The Support Vector Machine had an area under the curve of 0.9800 on the training set. The results of the model were exported based on 10-fold cross-validation. In addition, the area under the curve on the test set is 0.70. The external validation proves the reliability of the prediction model.

**Conclusion:**

A support vector machine based on perinatal factors was developed to predict the occurrence of neurodevelopmental impairment in preterm infants with gestational age  < 32 weeks. The prediction model provides clinicians with an accurate and effective tool for the prevention and early intervention of neurodevelopmental impairment in this population.

## Introduction

The newborn comes from a homeostatic condition, which is characterized by warmth, darkness, quiet, and protection. On the contrary, the extrauterine world, even when it is strictly controlled and kept under optimal conditions, is characterized by handling, cold, noise, and light. Light as a potential stimulus for the activation of the central nervous system at human birth *via* the melanopsin-dependent retinal non-visual pathway. Light may be considered to be the main trigger of a sudden shift of the brain from a prenatal pattern of functions to a neonatal setup, thereby activating the first breath (Polese et al., 2022). Evidence of a number of neurological events occurring before first breath opens the way to the primacy of the Central Nervous System (CNS) at birth, due to an “extrauterine” activation induced by a necessary but still undefined specific quid in the environment. This activation is expected to set the basis for the occurrence of all those specific physiological conditions that lead to events determining the influx of air in the lungs, first breath and continuous and successful extra-uterine breathing (Polese et al., 2021). In premature infants, connections involving superior temporal, hippocampal, and occipital areas, among others, were stronger compared to fetuses. Premature infants showed stronger connectivity in sensory input and stress-related areas suggesting that extra-uterine environment exposure alters the development of select neural networks in the absence of structural brain injury. Early extrauterine exposure alters functional brain connectivity in premature infants without structural brain injury (De Asis-Cruz et al., 2020). Relative to *in-utero*, the extrauterine environment increases exposure to noise, light, handling and clinically painful procedures, as well as pharmacological interventions, which all have the potential to detrimentally influence developmental trajectories. Premature birth and early exposure to the extrauterine environment can result in widespread neurodevelopmental impairment, dependent on the degree of prematurity at birth and postnatal insults (Schmidt Mellado et al., 2022). Neurodevelopmental impairment (NDI) can cause motor delays, cognitive delays, language delays, blindness, deafness and, in severe cases, cerebral palsy, which is a main cause of poor quality of life of preterm infants. Prematurity is an important factor that can lead to NDI. The incidence of childhood developmental disabilities in China was 6,654 per 100,000 in 2016, of which 70–80% of cerebral palsy is associated with prenatal factors including prematurity (Rehabilitation CsRCoCMAo, 2015). Globally, approximately 5–11% of newborns are born prematurely, and preterm infants born <32 weeks accounts for approximately 15% of preterm births. In China, the preterm delivery rate is 6.9% which has the second highest absolute amount in the world (1.16 million per year) (Chawanpaiboon et al., 2019). Preterm infants with gestational age < 32 weeks are more likely to have severe complications, including IVH, infectious diseases, BPD, ROP etc., which are closely related to NDI (Zhang et al., 2017; Jin et al., 2022). In a recent meta-analysis including 10,293 very-low-birth-weight infants or infants born with gestational age < 32 weeks, the prevalence of cognitive delay, motor delay and cerebral palsy was 14.3, 16.4 and 4.5%, respectively (Pascal et al., 2018). Furthermore, 28–40% of the sample exhibit symptoms of neurodevelopmental impairment, such as cerebral palsy, intellectual disability, visual and hearing impairments, varying degrees of cognitive, and behavioral and mental disorder at 1 to 3 years of age (Godeluck et al., 2019). Early identification and timely intervention for NDI can improve the outcomes of preterm infants who are prong to NDI, and subsequently improve their quality of life and reduce social burden. Prediction models for bronchopulmonary dysplasia (BPD), and necrotizing enterocolitis (NEC)have been reported and have shown satisfying predictive accuracy (Nino et al., 2020; Wang et al., 2022). However, very few studies focus on the prediction model for the development of NDI in preterm infants. The objective of this study was to set up a risk model for short-term neurodevelopmental impairment in preterm infants born before 32 weeks of gestation, and to provide clinicians with an early prediction tool, based on which timely intervention can be applied to improve the outcomes of this population.

## Methods

### Study design and population

This is a retrospective study and has been approved by the Ethics Committee of The Maternal and Child Health Hospital of Guangxi Zhuang Autonomous Region [(2019–4) NO 4.]. Eligible preterm infants hospitalized in the Maternal and Child Health Hospital of Guangxi Zhuang Autonomous Region between September 1st, 2019 and March 1st, 2021 were enrolled. A prediction model was built based on this sample. According to the international common practice, the training set and test set are divided according to 8:2 randomly by Microsoft Excel. Inclusive criteria were as follows: 1. Gestational age less than 32 weeks; 2. Have completed the follow-up assessment at 18 months corrected age. Exclusion criteria were as follows: 1. Central nervous system infection; 2. Major congenital malformations; 3. Hereditary endocrine and metabolic diseases. 4. Discharged against medical advice or died at discharge; 5. Incomplete record data.

### Data collection

Data regarding the general information of the infants and their parents, perinatal conditions of the infants (including sepsis, IVH, NEC, ROP, BPD and so on), and the neurodevelopmental assessments at 18 months corrected age. We used Gesell Developmental Scale and Sign–Significate (S-S) relations combining with conical manifestations to assess motor, language, cognitive, and individual social development.

### Model building

Model building and evaluation: by using multiple logistic regression model and Support Vector Machine (SVM), a prediction model for NDI in preterm infants born <32 weeks was constructed. The variables with significant effects on NDI (95% confidence level) were screened out by the multivariate logistic regression model, and these indicators were used as important references for subsequent model building.

For a given training set of samples D, the $x_{i}$ are input variables containing various types of feature values, where i = 1, 2…, m。


$$D=\left\{\left(x_{1}{,y}_{1}\right),\left(x_{2}{,y}_{2}\right),\ldots ,\left(x_{m}{,y}_{m}\right)\right\},y_{i}=\left\{1,1\right\}$$

The generalized form of the above linear equation is as follows.


(1) $$\omega^{T}x+b=0$$

Where $\omega =\left(\omega_{1};\omega_{2};\ldots ,\omega_{d}\right)$ is the normal vector of the above linear equation, the normal vector is always perpendicular to the direction of the tangent to the linear equation, and the division of the hyperplane is denoted as (ω,b).

It is easy to see that the Euclidean distance from any point x in space to the hyperplane can be written as:


(2) $$r=\frac{\left|\omega^{T}x+b\right|}{║\omega ║}$$

For correctly classified samples, the following relationship always holds.


(3) $$\{\begin{array}{c} \omega^{T}x+b\geq +1,y_{i}=+1;\\ \omega^{T}x+b\leq +1,y_{i}=-1. \end{array}$$

The interval is calculated as follows.


(4) $$\gamma =\frac{2}{║\omega ║}$$

where, ω is two norm.

The problem of dividing the hyperplane is transformed into the problem of maximizing the interval, that is, finding the parameters that can satisfy the constraint under (ω,b), maximizes the interval, i.e.,


(5) $$\begin{array}{l} \;\;\;\;\;\;\;\;\;\;\;\;\;\;\;\;\;\;\;\;\;\;\;\;\;\;\;\;\;\;\;\;\;\underset{\omega ,b}{\max}\frac{2}{║\omega ║}\\ s.t.y_{i}\left(\omega^{T}x_{i}+b\right)\geq 1,i=1,2,\ldots ,m. \end{array}$$

After the programming problem is solved, receiver operating characteristic curve (ROC) and the area under curve (AUC) were used to assess the predictive accuracy and consistency of the model. Both discrimination and calibration were assessed by a bootstrap method with 505 resamples.

### Statistical analysis

In this study, the random partitioning of the training and test sets was performed by using Microsoft Excel. All the predictions for the samples were made using the MATLAB classification model tool. The F1 score, confusion matrix, AUC, and the importance level of each indicator were calculated by the MATLAB codes, including confusionchart, statsOfMeasure, etc.

### Definitions

Birth weight was defined as the weight of the newborn within 1 h after birth (Shao and Qiu, 2019). Intraventricular hemorrhage (IVH) is diagnosed and graded according to Papile’s criteria (Lu-Ann Papile et al., 1978). Adequate prenatal glucocorticoids defined as glucocorticoids administered to the mother at least 48 h before delivery (betamethasone 12 mg q24h intramuscularly or dexamethasone 6 mg q12h) (Sweet et al., 2019). The diagnostic criteria for neonatal diseases such as neonatal asphyxia, small for gestational age, neonatal sepsis, respiratory distress syndrome (RDS), retinopathy (ROP). NEC was defined according to Bell’s criteria (Walsh and Kliegman, 1986). Definitions of pregnancy induced hypertension syndrome (PIH), intrahepatic cholestasis of pregnancy (ICP), gestational diabetes mellitus (GDM), premature rupture of membrane (PROM) and cardiac vascular dysfunction are to be found in Obstetrics (Xie and Xing, 2013). Chorioamnionitis was diagnosed clinically, the diagnosis was made when maternal fever (temperature > 38°C) combined with any of the following elements: maternal tachycardia (heart rate > 100 bpm), fetal tachycardia (fetal heart rate > 160 bpm), uterine tenderness, foul smell of amniotic fluid, elevated maternal white blood cell (WBC) count (Jianping, 2014). NDI refers to moderate to severe cerebral palsy, cognitive or motor scores below 2 standard deviations from normal, bilateral hearing impairment requiring hearing aids or bilateral blindness (Cao, 2013).

## Results

### General characteristics

**Figure 1:**
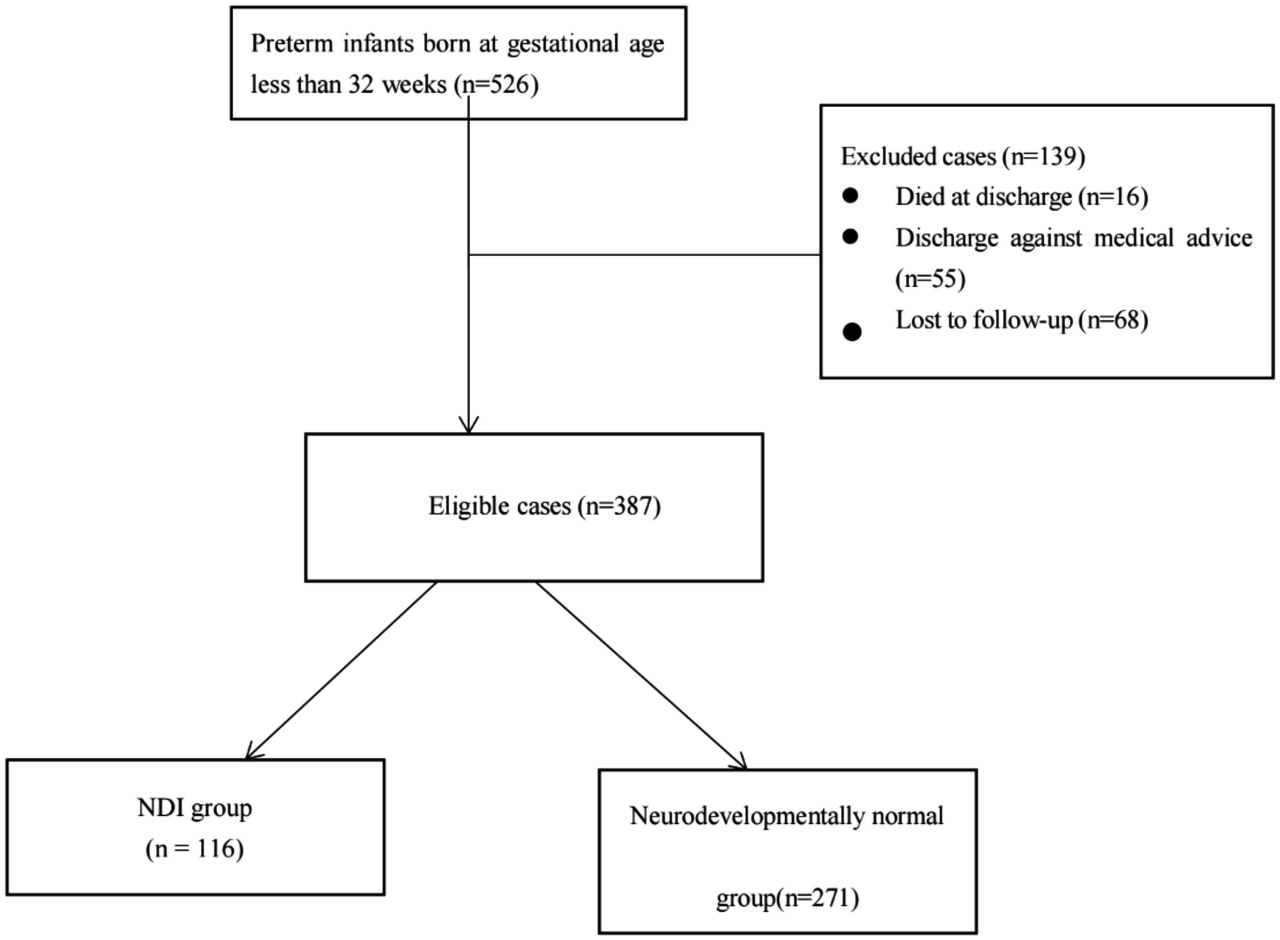
Flowchart of participants through each stage of the study. NDI: Neurodevelopmental impairment.

A total of 526 eligible preterm infants were enrolled in the study. Among them, there were 16 deaths, 55 infants were discharged against medical advice, 455 survived and were discharged from NICU. 68 lost to follow-up. Finally, 387 preterm babies were included in the study (Figure 1). The 387 children were divided into the NDI group (116) and the normal neurodevelopment group (271) according to the follow-up assessments.

### Dimensionality reduction

**Figure 2:**
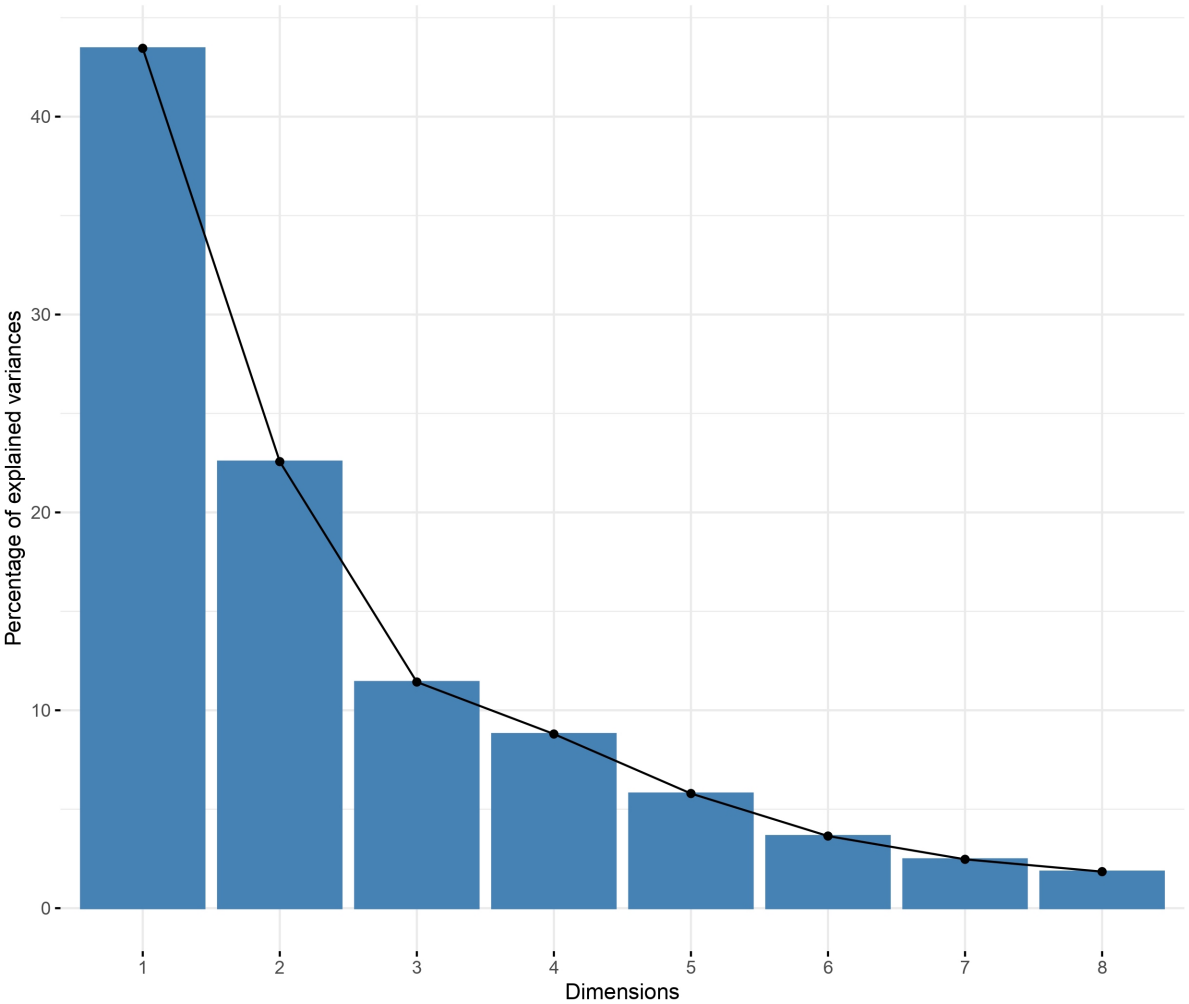
Principal component analysis Explained variance by dimension Using the maximum explained variance as a criterion, the horizontal axis is the individual dimensions and the vertical axis is the explained variance. It can be seen that the explained variance of the first dimension reaches more than 40 percent, the explained variance of the second dimension has more than 20 percent, and the explained variance of the first three dimensions reaches more than 75 percent.

**Figure 3:**
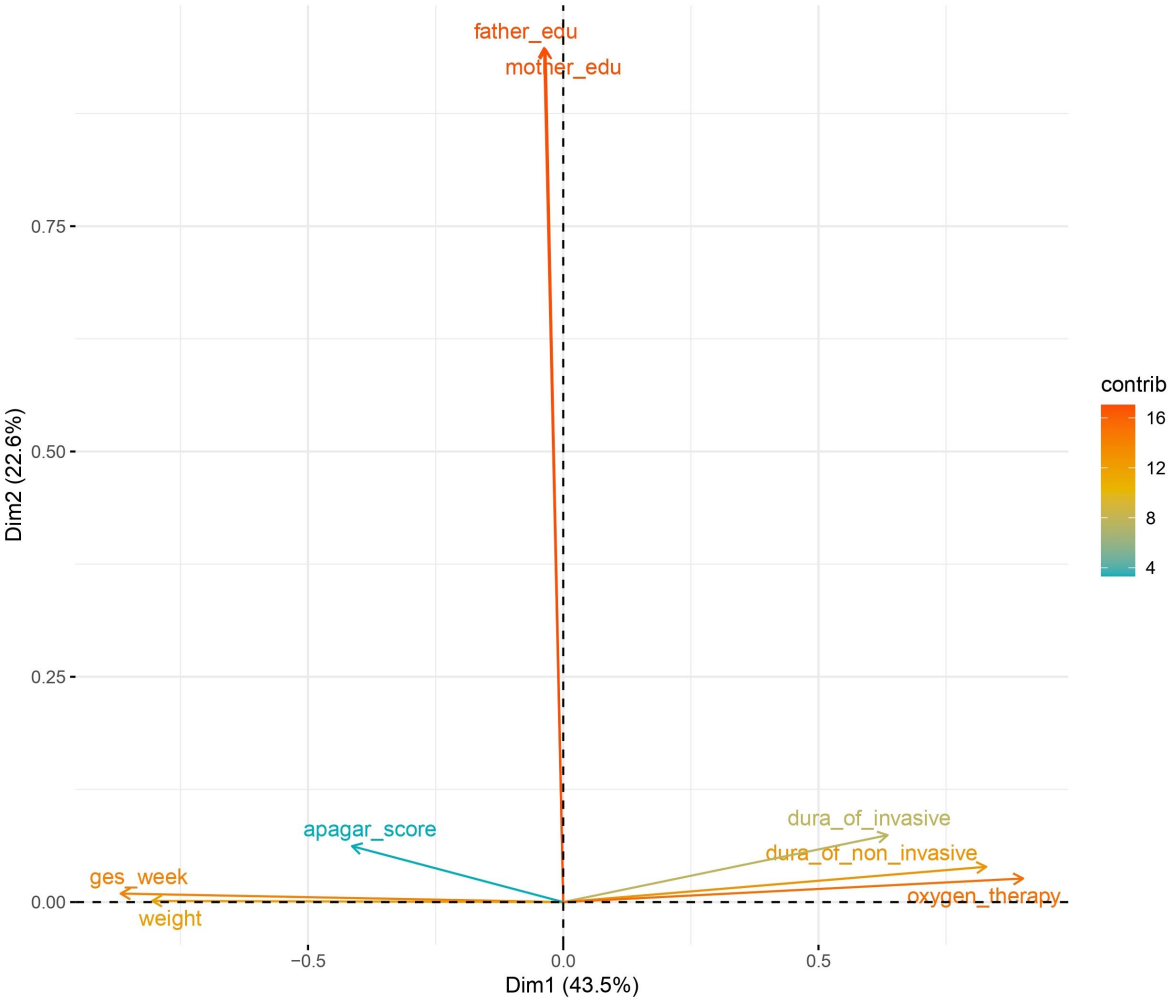
Numerical variables in the two-dimensional plane (principal component analysis) In the two-dimensional plane, you can see those 6 variables such as Gestational week, weight, etc. are mapped to the horizontal dimension more, mother’s education and father’s education were mapped to a greater extent to the vertical plane.

A principal component analysis was performed on the numeric variables (Figure 2), setting the maximum explained variance above 75%, further reducing the dimensionality of the numeric variables to 3 dimensions. Further, the numerical variables were mapped to the 2-dimensional plane. Gestational week, weight, 5-min Apgar score, duration of invasive ventilation, duration of non-invasive ventilation, and total duration of oxygen supplementation were mapped to a greater extent to the horizontal dimension; correspondingly, mother’s education and father’s education were mapped to a greater extent to the vertical plane (Figure 3).

### Model tuning

The training data and the test data were input into the model separately. The results of the model were exported based on 10-fold cross-validation (the proportion of validation set is 10%). Feature selection was performed on the variables refer to the results of logistic regression. The principal component analysis method was used to further reduce the dimensionality by setting the maximum explained variance as 75%, the model with better performance measures is selected.

### Screening for predictive factors

The multivariate logistic regression demonstrated that seven indicators were more important to predict the occurrence of NDI in preterm infants, including gestational age (*p* = 0.0004), extrauterine growth restriction (EUGR) (*p* = 0.0367), vaginal delivery (*p* = 0.0009), hyperbilirubinemia (0.0015; Table 1).

**Table 1:** Multivariate logistic regression analyses for screening predictors.

Variables	Estimate	SE	tStat	*P*
Intercept	10.7181	2.9037	3.6912	0.0002
Gender	0.0511	0.2235	0.2288	0.8191
Gestational week	−0.3532	0.0996	−3.5449	0.0004
Weight	−0.0008	0.0005	−1.5641	0.1178
5-min Apgar score	0.0958	0.0987	0.9703	0.3319
Intrauterine growth restriction	0.4594	0.3415	1.3452	0.1786
Extrauterine growth restriction	0.4835	0.2314	2.0893	0.0367
Vaginal delivery	0.6926	0.2081	3.3283	0.0009
Maternal age over 35	−0.2540	0.2296	−1.1065	0.2685
Mother’s education	−0.2083	0.3189	−0.6532	0.5137
Father’s education	0.0414	0.3224	0.1283	0.8979
Assistant reproduction	0.0508	0.2900	0.1752	0.8609
Gestational hypertension/diabetes	0.1476	0.2558	0.5770	0.5639
Premature rupture of membranes	−0.1507	0.2247	−0.6708	0.5024
Chorioamnionitis	0.3504	0.4152	0.8440	0.3987
Antenatal steroids	0.0375	0.2823	0.1329	0.8943
Neonatal respiratory distress syndrome	−0.0890	0.2379	−0.3741	0.7083
Bronchopulmonary dysplasia	−0.4907	0.2571	−1.9086	0.0563
Intracranial hemorrhage greater than or equal to grade 3	0.7908	0.4349	1.8185	0.0690
Patent ductus arteriosus	0.0348	0.2298	0.1513	0.8797
Hyperbilirubinemia	1.2734	0.4017	3.1701	0.0015
Cholestasis	0.2702	0.2384	1.1334	0.2570
Anemia	−0.3713	0.3186	−1.1656	0.2438
Fungal infections	−1.3419	0.8638	−1.5534	0.1203
Hypoglycemia	0.0178	0.3059	0.0582	0.9536
Hypothyroidism	0.4903	0.8274	0.5926	0.5535
Retinopathy of prematurity	−0.2308	0.3323	−0.6945	0.4874
Duration of invasive ventilation	0.0032	0.0210	0.1512	0.8799
Duration of non-invasive ventilation	0.0030	0.0133	0.2298	0.8182
Total duration of oxygen supplementation	−0.0172	0.0101	−1.7077	0.0877
Neonatal sepsis	0.0127	0.2397	0.0529	0.9579

### ROC plotting

**Figure 4:**
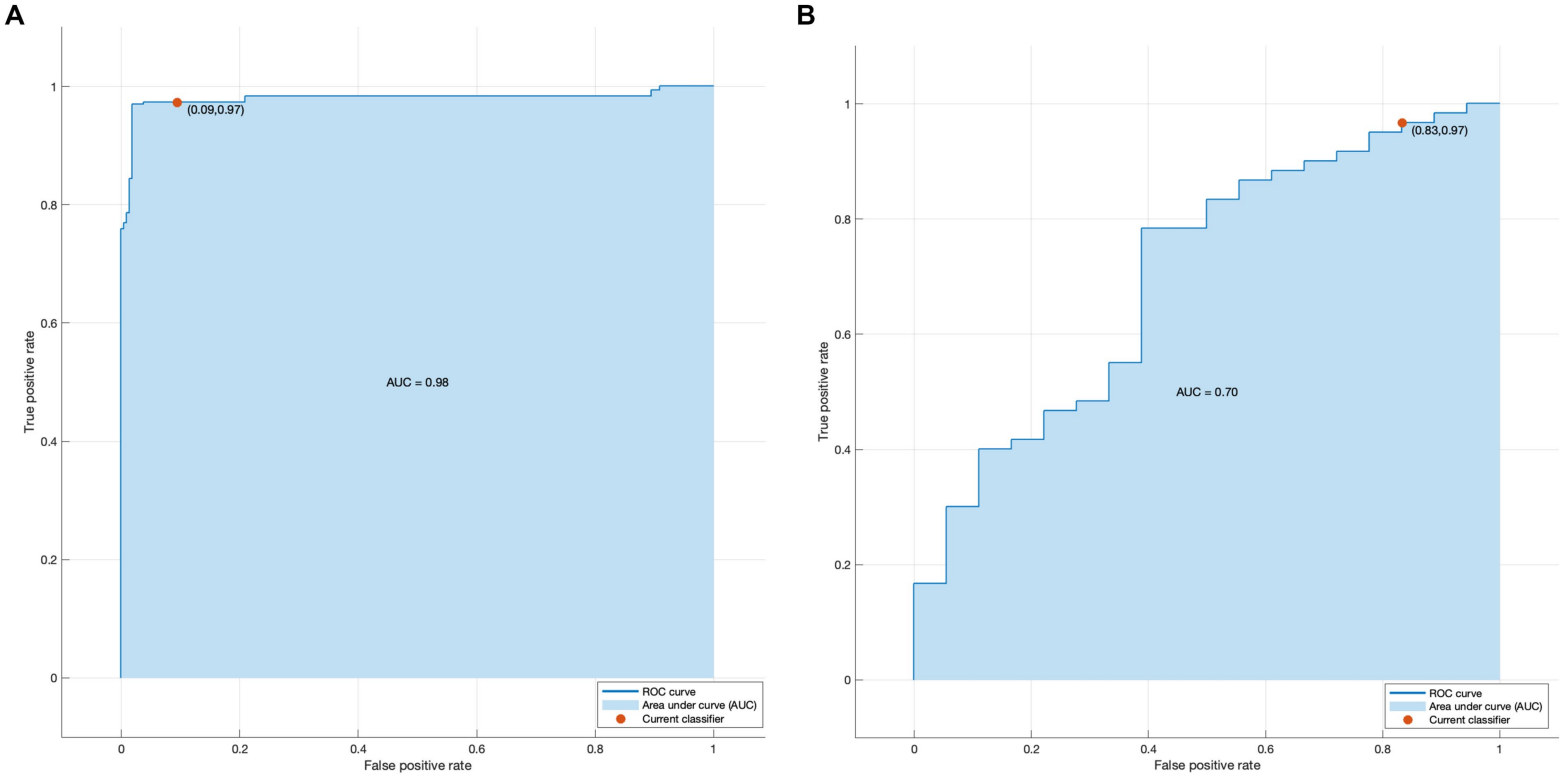
**(A)** Receiver operator characteristic curve for the training set **(B)** Receiver operator characteristic curve for the test set.

Based on the true classification of the training set and the predicted classification under the SVM, making an ROC curve of whether a preterm infant is NDI, where 1 indicates “positive class” and 0 indicates “negative class.” Figure 4 shows that the AUC of the training set (Figure 4A) and the test set (Figure 4B) were 0.98 and 0,70, respectively. The prediction results and interpretability of the model are good.

### Risk prediction

**Figure 5:**
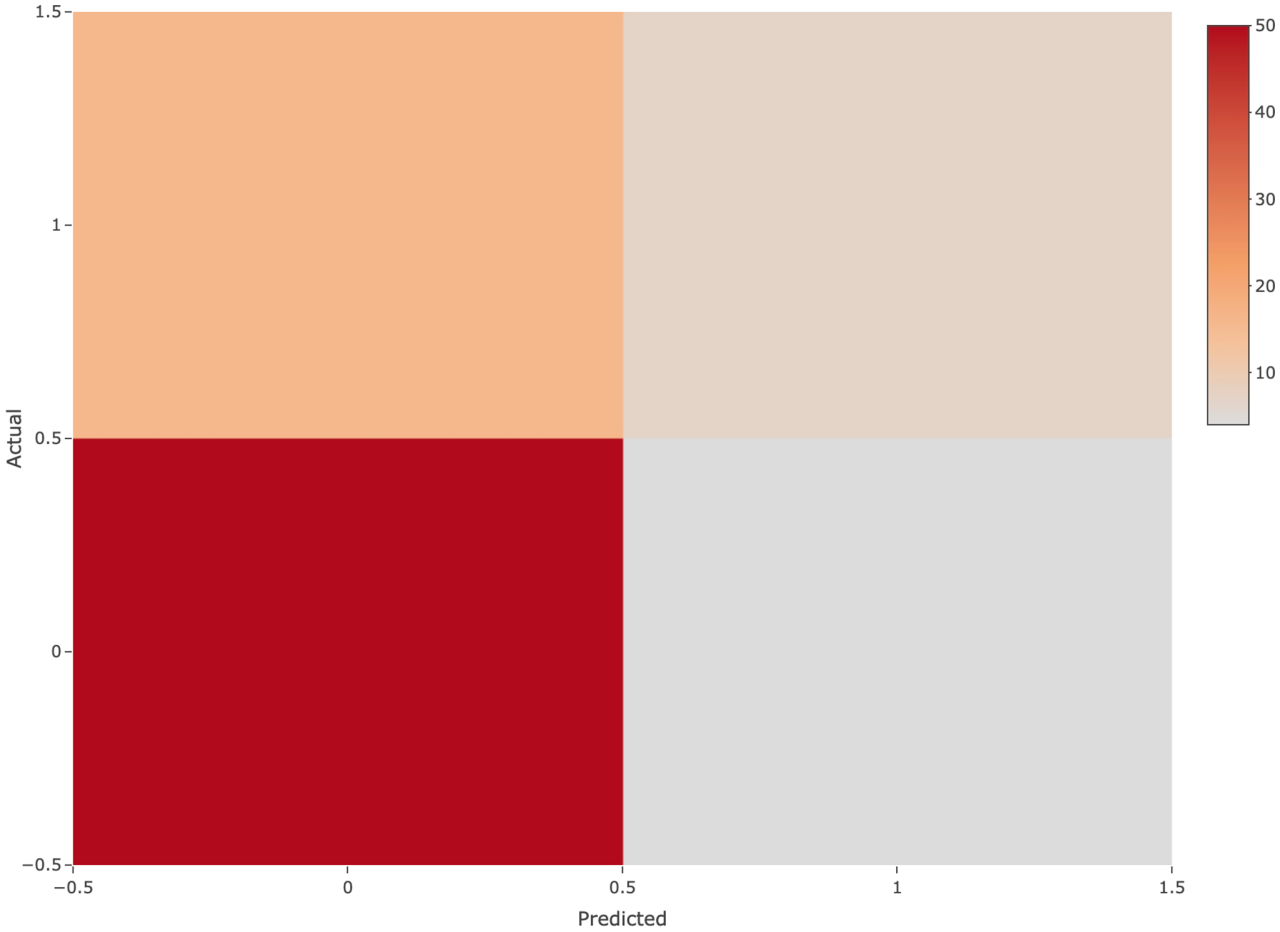
Confusion matrix The horizontal axis represents the predicted class, the vertical axis represents the true class, 1 represents the positive class, 0 represents the negative class.

The accuracy and AUC of the SVM built from above metrics on the training set were 94.5% and 0.9800. In addition, the results of the confusion matrix analysis showed that the predictive effect of the model was good. These data showed that the predictive model had great potential for clinical decision making (Figure 5).

### Compare with other models

When compare the accuracy and AUC of the SVM with other main strain machine learning models, the SVM is both ranked first, which to some extent, demonstrates the correctness of the selection of the SVM model as a classifier (Table 2).

**Table 2:** The accuracy and AUC of machine learning models.

Model	Area under the curve
Support vector machine	0.70
Multivariate logistic regression model	0.60
Radom forest	0.69
Neural network	0.70
Naïve Bayes	0.59

## Discussion

This study is the first to use machine learning method combined with perinatal factors to build a prediction model for NDI. For preterm infants with gestational age < 32 weeks, the higher the value of the probability that the prediction model is biased towards the positive category, the higher the risk of NDI. It revealed that gestational age, EUGR, vaginal delivery, hyperbilirubinemia were predictors for NDI. The ROC curve on the test set shows that the effect and the interpretability are good. The AUC of the training set and the test set were 0.98 and 0.70, respectively. In addition, the results from the confusion matrix analysis demonstrate that the model has a good predictive effect. The external validation proved the reliability of the prediction model (Table 2).

The younger gestational age, the higher risk of brain developmental disorders they face (Burnett et al., 2018). In a retrospective multicenter study of 307 preterm infants born with gestational age < 32 weeks in 10 tertiary hospitals in Guangxi, we found that the rate of neurodevelopmental impairment was inversely corelated with gestational age (Li et al., 2019). This study also found that gestational age was predictor of NDI.

Our study demonstrated that delivery mode was also a predictor of NDI. A study in China found that vaginal delivery was an independent risk factor for brain injury in preterm infants born with gestational age < 34 weeks (Arigonggaowa and Bai, 2018). The reasons might be the fact that the fetal cranium was compressed during vaginal delivery and the intracranial venous pressure was significantly increased, which easily caused bleeding from ruptured capillaries. However, an epidemiological research of brain injury in Jiangsu, China showed, a higher rate of cerebral infarction in neonates delivered through C-section (Neonates JSDaTRCGoBli, 2019).

EUGR is also one of the predictors of NDI in this study. Nutrition is positively associated with weight gain, increased brain volumes and white matter maturation on magnetic resonance imaging at term equivalent age and neurodevelopment in infancy. Weight gain, linear and head circumference growth are all markers of nutritional status and are independently associated with long-term neurodevelopment (Skinner and Narchi, 2021). In preterm infants, neurodevelopment is very sensitive to nutrition in the first few weeks of life and the impact of nutritional deficiencies may persist well into the eighth years of age (Coviello et al., 2018). Complications of prematurity, such as BPD, feeding intolerance, NEC et al. would all contribute to inadequate supply or impaired intake of nutrition, ultimately leads to EUGR (Makker et al., 2021). The previous multicenter study by our study team found that the incidence of EUGR in extremely-premature infants was as high as 69.7% (Liping et al., 2019). It was reported that EUGR was significantly associated with a low mental development index (MDI) at 24 months of age (Chien et al., 2018).

Bilirubin-induced brain injury in the neonatal period has detrimental effects on neurodevelopment that persist into childhood and adulthood, contributing to childhood developmental disorders. In this study, hyperbilirubinemia is also a predictor of NDI in preterm infants. Unconjugated bilirubin is a potent antioxidant that may be useful for protecting against oxidative injuries, but it becomes a potent neurotoxin once it crosses the blood brain barrier. Free bilirubin can pass through the blood brain barrier and trigger a neuroinflammatory response in neonatal hyperbilirubinemia (Amin et al., 2019). Activation of neuroinflammation in the early life will affect fetus/infant neurodevelopment (Han et al., 2021). The blood brain barrier permeability is higher in preterm infants, especially when combined with acidosis or sepsis, etc. Excessive/prolonged neuroinflammation activated by bilirubin will interfere with development of the immature brain and lead to neurological sequelae (Zhang et al., 2023).

The incidence of BPD in preterm infants born with gestational age < 32 weeks and had a birth weight < 1,500 g was as high as 91.18% (Yao et al., 2017). As Gu et al. (2020) reported, children with BPD have a higher rate of delayed neurobehavioral development in the first year of life compared with non-BPD children. According to Malavolti et al. (2018) severe BPD was an independent predictor of NDI. However in our study BPD was not the predictor of NDI, there was a trend that the NDI group had a higher rate of BPD. Thus, in the management of preterm infants, the respiratory management should afford enough attention.

Maternal factors are closely related to the condition of newborn. Maternal education and other socioeconomic issues are strongly associated with some adverse perinatal outcomes, including preterm birth, low Apgar score, cerebral distress, respiratory distress, and small for gestational age (Cantarutti et al., 2017). Neonatal complications are more likely to occur in the newborns of less educated mothers, probably due to their neglect of prenatal care and pregnancy complications (Clapp et al., 2020). Maternal education was not a predictor in this present study, however, gestational age and EUGR which may result from poor maternal educational status were proved to be predictors of NDI in the study population.

Recently, there has been an appreciation of the importance of environmental factors in long-term outcomes of preterm babies. In intensive care, premature infants are exposed to stressors and stimuli including diseases, medications, painful procedures, and noise and light at a time when they are not able to be with their parents and are not developmentally ready. Unsuitable conditions in the environments where an infant receives long-term treatment and care can have a negative effect on the infant’s nervous system. Developmental care comprises a number of different activities such as reducing light and sound and a range of care activities, including positioning, kangaroo care, swaddling of the infant, and non-nutritional sucking which promote the growth and development of preterm infants during and after the intensive care process experienced in the NICU. Positive parenting and parents’ mental health are shown to have long lasting advantages for preterm infants (Maccari et al., 2017; Cheong et al., 2020; Kucuk Alemdar and Inal, 2020). Developmental care was a routine practice for preterm infants in the study center, therefore it was not included in the analysis.

As the advancement of the care for preterm infants, the rate of white matter injury is extremely low in our center, therefore, we did not include white matter injury in the multivariate logistic regression. Similarly, the rate of severe IVH was low in both groups, the multivariate logistic regression did not show significant difference. Previous study showed that in addition to regular intermittent kangaroo mother care, delivery room skin-to-skin contact (DR-SSC) promotes mother–child interaction and decreases risk of maternal depression and bonding problems. Thus, DR-SSC may have positive effects on preterm development (Mehler et al., 2020). Immediate skin- to- skin contact for the first six postnatal hours had beneficial effects on the cardiorespiratory stabilization of very preterm infants. Thus, immediate skin-to-skin care is feasible and may be the desirable standard of care for very preterm infants (Linnér et al., 2022). Unfortunately, skin-to-skin care immediately after birth in delivery room have not been carried out in our center, thus was not included in the analysis.

### Strengths and limitations

This present study built an NDI prediction model using SVM machine learning methods by establishing a birth cohort of preterm infants less than 32 weeks of gestation. This method effectively solves the problem of model prediction bias under high-dimensional data with small samples. There are several limitations to this study. First, pain assessment, family income, parents’ mental health, environmental factors such as parenting, light and noise were not included in the study due to data unavailable, which may cause potential bias. Second, in this study, data was collected from a single center, which could only be considered representative of the population of south China; therefore, we will seek to carry out an external validation assessment in a multi-center study.

In conclusion, A support vector machine based on perinatal factors was developed to predict the occurrence of neurodevelopmental impairment in preterm infants with gestational age < 32 weeks. The prediction model provides clinicians with an accurate and effective tool for the prevention and early intervention of neurodevelopmental impairment in this population.

## Data availability statement

The original contributions presented in the study are included in the article/supplementary material, further inquiries can be directed to the corresponding author.

## Ethics statement

The studies involving human participants were reviewed and approved by Ethics Committee of the Maternal and Child Health Hospital of Guangxi Zhuang autonomous region. Written informed consent from the participants’ legal guardian/next of kin was not required to participate in this study in accordance with the national legislation and the institutional requirements.

## Author contributions

YL conceptualized and designed the study, collected and analyzed data, checked literature, and drafted the manuscript. ZZ contributed to statistical analysis and literature review. QW and YS interpreted data and checked literature. YM collected data, drafted the manuscript and implemented the study. LJ, WL, and ML collected data, drafted the manuscript, LZ, XL, and DM verified the underlying study data. All authors had full access to all the data in the study and accept responsibility to submit for publication. All authors revised the manuscript and approved the final manuscript as submitted.

## Funding

National Key Research and Development Program of China (2022YFC2704803); Guangxi Clinical Research Center for Pediatric Diseases (桂科AD22035121). Guangxi Natural Science Foundation Project (2019GXNSFBA185040). Guangxi Medical and Health Appropriate Technology Development, Promotion and Application Project (S2021074). Guangxi Medical and Health Appropriate Technology Development, Promotion and Application Project (S2017058).

## Conflict of interest

The authors declare that the research was conducted in the absence of any commercial or financial relationships that could be construed as a potential conflict of interest.

## Publisher’s note

All claims expressed in this article are solely those of the authors and do not necessarily represent those of their affiliated organizations, or those of the publisher, the editors and the reviewers. Any product that may be evaluated in this article, or claim that may be made by its manufacturer, is not guaranteed or endorsed by the publisher.
